# Treatment of Single or Multiple Brain Metastases by Hypofractionated Stereotactic Radiotherapy Using Helical Tomotherapy

**DOI:** 10.3390/ijms15046910

**Published:** 2014-04-22

**Authors:** Aiko Nagai, Yuta Shibamoto, Masanori Yoshida, Koichi Wakamatsu, Yuzo Kikuchi

**Affiliations:** 1 Radiation Therapy Center, Fukui Saiseikai Hospital, 7-1, Funabashi, Wadanaka-cho, Fukui 918-8503, Japan; E-Mails: mas-yoshida@fukui.saiseikai.or.jp (M.Y.); y-kikuchi@fukui.saiseikai.or.jp (Y.K.); 2 Department of Radiology, Nagoya City University Graduate School of Medical Sciences, Nagoya 467-8601, Japan; E-Mail: yshiba@med.nagoya-cu.ac.jp; 3 Department of Neurosurgery, Fukui Saiseikai Hospital, 7-1, Funabashi, Wadanaka-cho, Fukui 918-8503, Japan; E-Mail: k-wakamatsu@fukui.saiseikai.or.jp

**Keywords:** brain metastases, stereotactic radiotherapy, helical tomotherapy

## Abstract

This study investigated the clinical outcomes of a 4-fraction stereotactic radiotherapy (SRT) study using helical tomotherapy for brain metastases. Between August 2009 and June 2013, 54 patients with a total of 128 brain metastases underwent SRT using tomotherapy. A total dose of 28 or 28.8 Gy at 80% isodose was administered in 4 fractions for all tumors. The mean gross tumor volume (GTV) was 1.9 cc. Local control (LC) rates at 6, 12, and 18 months were 96%, 91%, and 88%, respectively. The 12-month LC rates for tumors with GTV ≤0.25, >0.25 and ≤1, and >1 cc were 98%, 82%, and 93%, respectively; the rates were 92% for tumors >3 cc and 100% for >10 cc. The 6-month rates for freedom from distant brain failure were 57%, 71%, and 55% for patients with 1, 2, and ≥3 brain metastases, respectively. No differences were significant. No major complications were observed. The 4-fraction SRT protocol provided excellent tumor control with minimal toxicity. Distant brain failure was not so frequent, even in patients with multiple tumors. The results of the current study warrant a prospective randomized study comparing single-fraction stereotactic radiosurgery (SRS) with SRT in this patient population.

## 1. Introduction

Patients with brain metastases have been traditionally treated by surgery, whole-brain radiotherapy (WBRT), single-fraction stereotactic radiosurgery (SRS), or hypofractionated stereotactic radiotherapy (SRT) [1–4]; in the past decade, the use of SRS has spread considerably. Recently published American Society for Radiation Oncology evidence-based guidelines on the management of newly diagnosed brain metastases state that patients presenting with multiple brain metastases (all less than 3–4 cm) have various treatment options, including SRS alone, WBRT with SRS boost, or WBRT alone, with no mention of SRT [5].

Many authors have reported the efficacy of SRS to obtain local tumor control [6–8]. However, hypofractionated SRT seems to become more appropriate than single-fraction SRS with the increase in tumor size, because fractionation provides a radiobiological advantage over single-fraction treatment [9]. As the tumor enlarges, the hypoxic fraction is considered to increase, so that the tumor becomes more radioresistant [10]. For tumors with a high hypoxic fraction, a single high radiation dose is not efficient enough. With fractionation, surviving hypoxic cells are expected to reoxygenate, so that they become more radiosensitive [11,12]. In addition, cell-cycle redistribution may also lead to increased radiosensitivity, although its contribution to radiosensitivity might be much less than that expected by reoxygenation [12]. Another biological advantage of fractionated SRT may derive from differences in the α/β ratio between normal tissues and tumors; it is usually assumed that the ratio is 2 to 3 Gy for normal brain tissues and up to 10 Gy for malignant tumors. Therefore, the use of lower radiation doses per fraction would increase the therapeutic ratio in the treatment of brain metastases, and SRT is recommended for tumors that are not expected to be controlled by a single tolerable dose of SRS. In fact, the local control (LC) rate for large tumors treated with SRS has been reported to be relatively low [13,14], and the clinical advantage of fractionated treatment on sensitive structures in the brain has been reported [9].

Recently, several authors have reported that helical tomotherapy could provide both SRS and SRT. In most studies, the outcomes of SRS/SRT combined with WBRT have been reported [15–20]. Only two studies have reported on SRS alone for brain metastases with respect to dosimetric equivalency between helical tomotherapy and intensity-modulated radiosurgery or gammaknife [19,20]. In this article, we report the results of our 4-fraction SRT protocol study using tomotherapy for brain metastases.

## 2. Results

### 2.1. Treatment Plan Analysis

The mean gross tumor volume (GTV) was 1.9 cc (range, 0.01–18). The median GTV was 0.4 cc and the median cumulative GTV was 1.9 cc. The averages of maximum dose and minimum dose for the planning target volume (PTV) were 34 (range, 28–39) and 27 (23–33) Gy, respectively. The homogeneity index (HI) was 1.2 ± 0.1 (mean ± SD, Table 1).

### 2.2. Local Control

**Figure 1.**
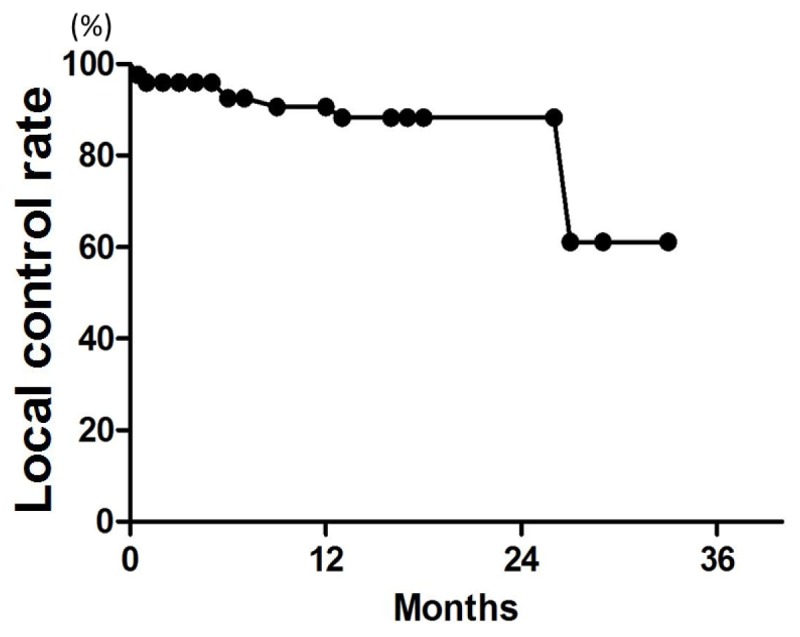
Local control (LC) curve for all 128 brain metastases.

**Figure 2.**
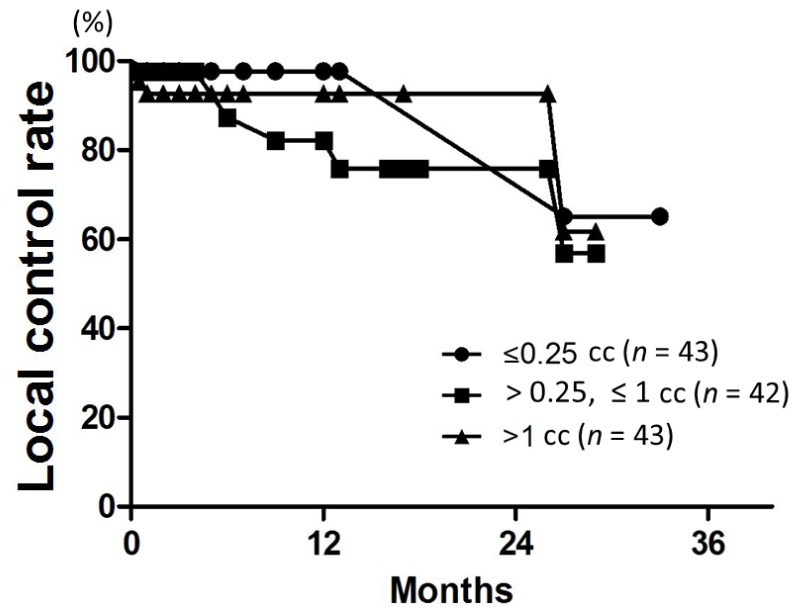
LC curves according to the gross tumor volume (GTV). There was no difference among the three groups (*p* = 0.4).

Patient characteristics are listed in Table 2. Patient age ranged from 38 to 89 years (median, 67 years). The median follow-up was 18 months for all patients (range, 3–34 months). LC rates at 6, 12, and 18 months were 96%, 91%, and 88%, respectively (Figure 1). On univariate analysis, the 12-month LC rate was 79% for patients with multiple metastases and 100% for those with single metastasis (*p* = 0.04) (Table 3). The 6-month LC rates for tumors with GTV ≤0.25, >0.25 and ≤1, and >1 cc were 98%, 87%, and 93%, respectively. One-year LC rates for GTV ≤0.25, >0.25 and ≤1, and >1 cc were 98%, 82%, and 93%, respectively, with no significant difference (Figure 2). The LC rate at 12 months was 92% for GTV >3 cc (*n* = 24), and 100% for GTV >10 cc (*n* = 6). On multivariate analysis, there was no significant factor in all subsets (Table 4). Table 5 shows the distribution of tumor size according to tumor histology. Most of the tumors >3 cc were moderately radiosensitive tumors like adenocarcinomas and squamous cell carcinomas.

### 2.3. Overall Survival

**Figure 3.**
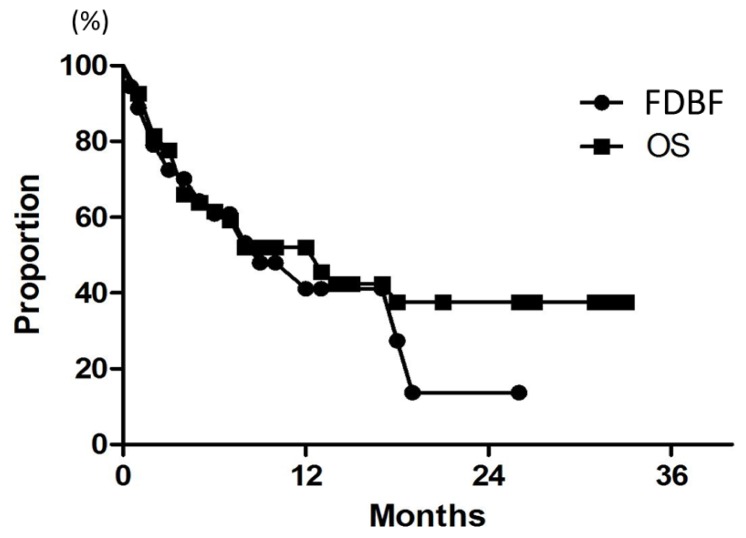
Curves for FDBF and OS for all 54 patients.

After SRT, 28 (52%) of the 54 patients eventually died of their disease. Median survival of all patients was 7 months. Overall survival (OS) rates at 6, 12, and 18 months were 61%, 52%, and 38%, respectively (Figure 3). The results of univariate analysis of relevant patient characteristics are shown in Table 3. Controlled extracranial disease was a significant predictor of OS when analyzed as a continuous variable (*p* = 0.0002). The 12-month OS was 31% for patients with total GTV >2 cc, but it was 72% for patients with smaller lesions (*p* < 0.0001). On multivariate analysis, only controlled extracranial disease was a significant predictor of OS, with a hazard ratio of 0.2 (*p* = 0.02) (Table 4).

### 2.4. Freedom from Distant Brain Failure

**Figure 4.**
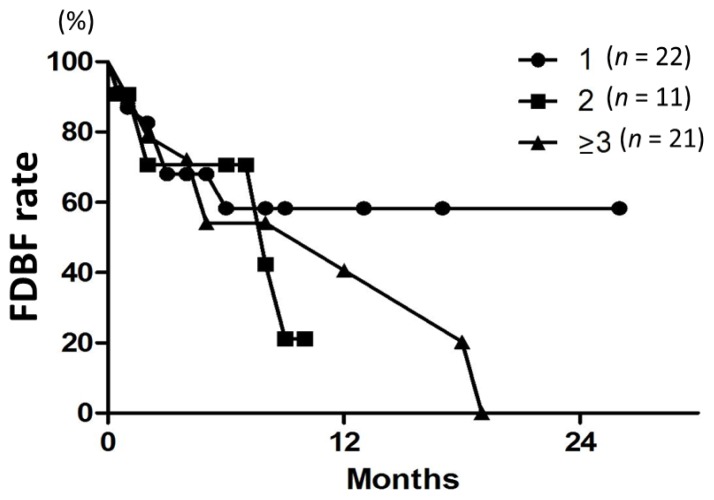
FDBF curves according to the tumor number (1, 2, or ≥3) in all 54 patients. There was no difference among the three groups (*p* = 0.7).

Median time to distant brain failure was 5 months. Freedom from distant brain failure (FDBF) rates at 6, 12, and 18 months were 61%, 41%, and 27%, respectively (Figure 3). The most common modalities for salvage were additional SRS (28%) and WBRT (9.3%). On univariate and multivariate analyses, there was no significant factor in all subsets (Tables 3 and 4). FDBF rates at 6 months for the number of brain metastases of 1, 2, and ≥3 were 57%, 71%, and 55%, respectively (Figure 4, *p* = 0.7).

### 2.5. Toxicity

There were no acute adverse events of grade 3 or higher. Only one patient had grade 2 nausea during treatment and one had grade 2 motor neuropathy. The late adverse events were grade 2 neurologic dysfunction observed in 2 patients (4%) and there was no radiation necrosis.

## 3. Discussion

In previous studies, single-fraction SRS for brain metastases yielded 12-month LC rates of about 80% [13,21]. A recent study reported the rate of 92%, but severe neurological complications (Radiation Therapy Oncology Group (RTOG) grade 3 and 4) occurred in 5.8% of the patients [22]. Data on cyberknife, with either SRS or SRT, suggested a 6-month LC of 80%–90% [23]. In our study using SRT alone, LC rates at 6 and 12 months were 96% and 91%, respectively. Thus, our fractionated SRT data compare favorably with the SRS and cyberknife data. In an SRS study, the LC rate decreased with increase in the tumor volume; for tumors >3 cc, the LC rate was approximately 60%, but for tumors >10 cc, it was only 25% [9]. In contrast, 12-month LC rates for GTV >3 and >10 cc were 92% and 100%, respectively, in the present study. The high LC rates for GTV >3 and >10 cc may in part be due to the small number of lesions (*n* = 24 and 6, respectively), but our study would suggest that LC rates of such tumors may be improved by using hypofractionated SRT. Tomotherapy has another advantage. Brain metastases often cause brain edema and bleeding, which result in dislocation of brain metastases. Megavoltage computed tomography (MVCT) images taken before each treatment session can detect the dislocation of a tumor, so it is possible to adjust the patient position for the precise delivery of irradiation to the tumor, which cannot be accomplished with cyberknife and older versions of linac-based SRT machines.

**Figure 5.**
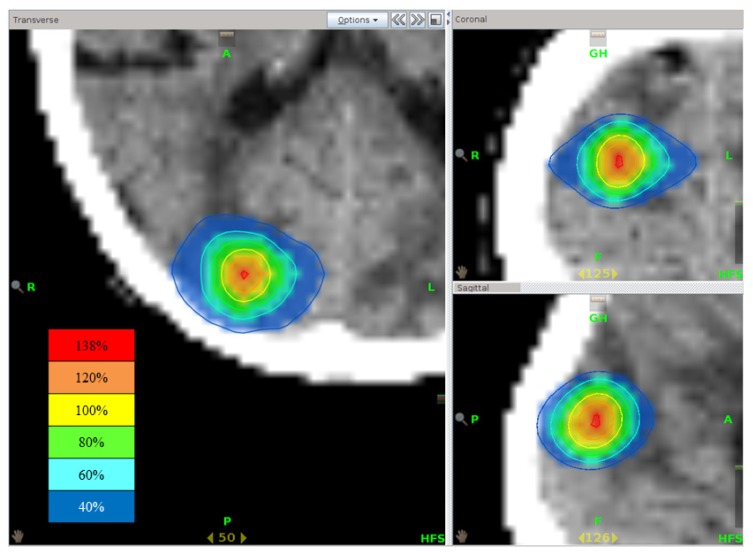
Isodose distribution of stereotactic radiotherapy using tomotherapy. The highest dose point is present at the central part of the brain metastasis.

**Figure 6.**
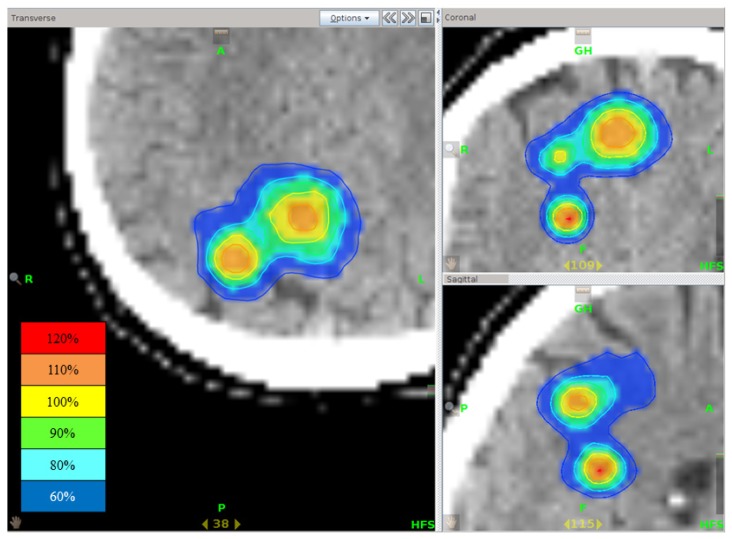
Isodose distribution of stereotactic radiotherapy using tomotherapy for multiple brain metastases. Although three tumors existed close to each other, rapid dose fall outside the PTV was seen.

Previous studies on SRT are shown in Table 6. Narayana *et al.* [24] reported on cyberknife SRT with a dose of 30 Gy in 5 fractions to the 100% isodose line. The LC rate was 70% at 12 months. Inoue *et al.* [25] reported SRT with 27 Gy in 3 fractions to the 60% isodose line; LC was obtained in 137 of 143 metastases (95.8%) during a median follow-up of 7 months. Fahrig *et al.* [26] used three different dose concepts for SRT: 5 × (6–7) Gy (A), 10 × 4 Gy (B), and 7 × 5 Gy (C). LC rates with A, B, and C regimens were 96%, 87%, and 85%, respectively. However, complications occurred significantly more often with A (22%) and C (7%) than with B (without complication). Therefore, they concluded that 10 × 4 Gy was well tolerated without severe adverse events. Martens *et al.* [27] analyzed clinical data using various dose concepts including reirradiation cases, and concluded that the equivalent dose in 2 Gy fractions of ≥35 Gy, which corresponds to the biological effective dose assuming an α/β ratio of 10 Gy (BED_10_) of ≥42 Gy, seemed to be most effective in patients with primary or recurrent limited brain metastases. The BED_10_ was 51.3 Gy for the 27 Gy/3 fr schedule and 56 Gy for the 40 Gy/10 fr schedule. Although the BED must be cautiously used in these dose-fractionation ranges [28,29], it was recommended that an appropriate BED_10_ range was 42 to 56 Gy. Therefore, we started with a prescription dose of 28 Gy delivered in 4 fractions, with a BED of 47.6 Gy. Furthermore, we intended to give higher maximum doses for the PTV than in the usual intensity-modulated radiation therapy (IMRT) [30]; the HI was 1.2 ± 0.1 in our study (Figure 5). Reported mean HI for coplanar IMRT radiosurgery, non-coplanar IMRT radiosurgery, and tomotherapy were 1.15 ± 0.05, 1.13 ± 0.04, and 1.18 ± 0.06, respectively, being lower than the HI in our study. The central part of tumors should contain hypoxic cells, so higher doses are necessary to kill such cells [31,32]. We assumed that the LC rates would improve by giving higher doses to the central parts. Moreover, rapid dose fall off outside the PTV was seen even in multiple brain metastases that existed close to each other (Figure 6). Since no severe acute and late complications were seen, still higher doses may be investigated in future studies, but the LC rates obtained in this study might be satisfactory for palliative purposes.

According to Likhacheva *et al.* [33], four factors were predictors of OS: (1) presence of extracranial disease at SRS; (2) total tumor volume of >2 cm^3^; (3) age > 60 years; and (4) baseline diagnosis-specific graded prognostic assessment (GPA). Antoni *et al.* [34] reported the following factors for better prognosis: (1) age under 60 years; (2) high Karnofsky performance status (KPS); (3) primary tumor control; (4) low number of extracranial metastases and brain metastases; and (5) triple negative subtype in breast cancer. In the current study, total GTV of >2 cc and the presence of extracranial disease were related to poor OS. Brain metastases are a heterogeneous population. The purpose of the GPA was to identify significant diagnosis-specific prognostic factors in a more recent era (1985–2007) compared with the RTOG recursive partitioning analysis (1979–1993). The original GPA was based on 4 criteria: age, KPS, number of brain metastases, and presence or absence of extracranial metastases [35]. In the current study, only the presence of extracranial disease was related to poor OS in multivariate analysis.

A Japanese multi-institutional prospective study (JLGK0901) [36] and Likhacheva *et al.* [33] reported a lack of prognostic significance of the number of brain metastases. In this study, treatment volume was more important than lesion number for predicting outcomes. In the European Organization for Research and Treatment of Cancer (EORTC) 22952–26001 study, WBRT reduced the 2-year relapse at the initial site of surgery from 59% to 27% (*p* < 0.001), and at new sites from 42% to 23% (*p* = 0.008) [37]. Aoyama *et al.* [6] reported that the 12-month actuarial rates of developing new brain metastases were 42% (95% CI, 24%–59%) in the WBRT + SRS group and 64% (95% CI, 49%–78%) in the SRS-alone group (*p* = 0.003). In the current study, the 12-month distant brain control rate was 41%. Our result compares unfavorably with those results obtained with the addition of WBRT. However, in the current study, the 6-month FDBF rates for the number of brain metastases of 1, 2, and ≥3 were 57%, 71%, and 55%, respectively, with no difference among the three groups. Therefore, the role of adding WBRT was unclear. Possibly, the spread of low-dose regions in the surrounding brain when treated with tomotherapy might contribute to the prevention of new lesion development. Although the percentage of the normal brain receiving 12 Gy (3 Gy × 4 fractions) was up to 12.4% (mean: 3.8%) in the present study, this percentage apparently increased in patients with multiple targets and a large tumor. According to the previous study [38], the maximum dose delivered to the brain is about 6.6 cGy in 4 fractions, so the influence of the MVCT dose appears to be little.

EORTC showed that the health-related quality of life (physical and role activities, and cognitive functioning at 8 weeks) of the patients who received adjuvant WBRT after surgery or SRS of a limited number of brain metastases was worse than that of patients in the observation-only group [39]. In addition, WBRT can cause brain atrophy [40]. Therefore, fractionated SRT using tomotherapy may be a suitable option in the treatment of single or multiple brain metastases. However, caution must be taken in planning the treatment. At frame-fixed, 2.4 mm thin-slice magnetic resonance imaging (MRI) for gammaknife surgery planning, an increased number of metastases were found in about one-third of the patients compared with the number on MRI at diagnosis [41]. Thus, thin-slice, preferably 1 mm slice, MRI is recommended before tomotherapy planning.

## 4. Methods and Materials

### 4.1. Study Design and Eligibility Criteria

This was a prospective protocol-based study approved by an institutional review board. Informed consent was obtained from all patients. The eligibility criteria were as follows: (1) brain metastases diagnosed with contrast-enhanced T1-weighted MRI; (2) maximum tumor diameter ≤3 cm; (3) tumor number ≤ 10; (4) no cerebrospinal fluid dissemination; (5) imaging findings and clinical presentation consistent with brain metastases; (6) no concurrent chemotherapy; and (7) no previous WBRT, SRS, or SRT to the target volume to be treated. Since the incidence of radiation necrosis is considered to increase with enlargement of the irradiated brain volume [25], we applied the 4-fraction regimen to tumors with a diameter ≤3 cm. We also treated larger lesions with more fractions, but they were not the subject of this study. The primary endpoint of the study was LC at 6, 12, and 18 months. Secondary endpoints were OS and FDBF at 6, 12, and 18 months, and the incidence of acute and late toxicities. For evaluation of these endpoints, at least 50 patients were scheduled to be accrued.

### 4.2. Patient Characteristics

All patients were treated at Fukui Saiseikai Hospital using helical tomotherapy (HT, Tomotherapy^®^, Accuray, Madison, WI, USA). Between August 2009 and June 2013, a total of 54 patients with 128 brain metastases matched the inclusion criteria.

### 4.3. Treatment Methods

All patients were immobilized in a supine position with a custom head-and-neck thermoplastic shell (Uni-frame, CIVCO Medical Solutions, Kalona, IA, USA), and a customized Head & Neck Vac-Lok Cushion (Med-Tec, Orange City, IA, USA) bag constructed for simulation and treatment. Planning computed tomography (CT) (Toshiba Medical Systems Corporation, Tochigi, Japan) images through the whole head and upper neck were obtained with a 2 mm slice thickness. Next, MRI was performed using a T1 3D FFE (Fast Field Echo) with gadolinium and 3D reconstruction of the axial, sagittal, and coronal planes with 1 mm slice thickness (thin-slice MRI). After scanning, the CT images were fused with MRI for delineation of the target and organs at risk. All target volumes and normal structures were contoured on the Pinnacle^3^ workstation (Philips Medical Systems, Madison, WI, USA). The GTV was equal to the clinical target volume (CTV). According to a previous single-fraction study [42], the localization error of tomotherapy using volumetric localization was 0.45 ± 0.17 mm, indicating a localization precision of 0.3 mm within a 95% confidence interval. In a multi-fraction study [43], the total systematic (and random) deviations reached 1.6 mm (0.9 mm), 1.7 mm (1.1 mm), and 1.1 mm (0.8 mm) for brain cancer patients in the medial-lateral, cranial-caudal, and anterior-posterior directions, respectively. Therefore, a 2 mm isotropic margin was used in principle for CTV for planning target volume (PTV) expansion in the present study, although the use of 2 mm margins is controversial [44]. The dose was 28 Gy at 80% isodose in 4 fractions delivered on consecutive weekdays. This dose was determined after trial and error based on the concept described in the Discussion. After confirming the safety of the dose, the daily dose was increased in July 2012 from 7.0 to 7.2 Gy.

Treatment plans were optimized in the tomotherapy planning software version 1, 2, 3, 4 (Accuray, Madison, WI, USA), with a convolution/superposition dose calculation algorithm. The planning parameters used for the HT plans were as follows: a field width of 1.0 cm, a pitch of 0.287, a modulation factor of 1.1–2.0, and a fine calculation grid (1.96 mm × 1.96 mm × slice thickness). All plans were verified in-phantom on the HT unit before treatment began. The *HI* was defined as the ratio of the maximum dose in the PTV (*D*_max_) and the prescription dose in the PTV (*D*_rx_):

(1) $$HI=D_{\text{max}}/D_{\text{rx}}$$

The patients were treated using the thermoplastic immobilization mask used for simulation, with positioning determined by coregistration of a MVCT scan acquired on the HT unit immediately before treatment. The initial automated MVCT co-registration using the bone and soft tissue setting on the HT unit was used with manual refinements by the therapists before treatment. An attending radiation oncologist verified all MVCT co-registrations.

### 4.4. Evaluation

Acute adverse reactions were monitored on the first and last days of treatment. Patients were followed at 1- to 3-month intervals after SRT and were evaluated by a physical examination and contrast-enhanced thin-slice MRI. The tumor response was evaluated by comparing follow-up MRI with pretreatment MRI and classified according to the Response Evaluation Criteria in Solid Tumors. Adverse reactions were evaluated using the Common Terminology Criteria for Adverse Events version 4.0.

### 4.5. Statistical Methods

LC, OS, and FDBF rates were calculated using the Kaplan-Meier method from the start of SRT. Univariate log-rank tests were used to assess the significance of prognostic factors affecting LC, OS, and FDBF. A two-sided *p* value of 0.05 or less was considered to reflect statistical significance. These univariate analyses were carried out using Prism (Graph Pad Institute Inc., San Diego, CA, USA). The Cox proportional hazard model was used for multivariate analysis to assess the effects of patient, tumor, and other factors on the endpoints. The forced entry test was used to assess the role of covariates in the model. These multivariate analyses were carried out using SPSS statistical software version 17.0 (SPSS, Inc., Chicago, IL, USA).

## 5. Conclusions

The 4-fraction SRT protocol with a total dose of 28 or 28.8 Gy provided excellent tumor control with minimal toxicity. Distant brain failure was not so frequent, even in patients with multiple tumors, possibly justifying the use of tomotherapy SRT in patients with multiple brain metastases. The results of the current study would warrant a prospective randomized study comparing single-fraction SRS with SRT in this patient population.

## Figures and Tables

**Table 1.** Tumor characteristics.

Characteristic	Number of tumors (%)		
Total number of patients	54		

Primary lesion		Histology	

Lung	46 (85)	Squamous	10 (22)
		Adeno	28 (61)
		Small	4 (9)
		Poorly differentiated	1 (2)
		Undifferentiated	3 (6)

Liver	1 (2)	Poorly differentiated	1

Unknown	1 (2)	–	–
Colon	1 (2)	Adeno	1

Breast	4 (7)	Ductal	3 (75)
		Scirrhous	1 (25)

Uterus cervix	1 (2)	Squamous	1

Total number of metastases		128	
Number of metastases, median (range)		2 (1–8)	
GTV (cc), median (range)		1.9 (0.01–18)	
PTV maximum (Gy), median (range)		34 (28–39)	
PTV minimum (Gy), median (range)		27 (23–33)	
HI, median (range)		1.2 (1.0–1.4)	

Abbreviations: GTV, gross tumor volume; PTV, planning target volume; and HI, homogeneity index.

**Table 2.** Patient characteristics.

Characteristic		Number of patients (%)
Total number of patients		54

Gender	Male	30 (56)
	Female	24 (44)

Age (years) median (range)		67 (38–89)

Extracranial disease	Yes	42 (78)
	No	12 (22)

Previous SRS or SRT *	Yes	38 (70)
	No	16 (30)

Previous surgery	Yes	4 (7)
	No	50 (93)

Recurrence after surgery	Yes	2 (50)
	No	2 (50)

Total dose at 80% isodose	28 Gy	33 (61)
	28.8 Gy	21 (39)

RPA class	I	7
	II	42
	III	5

GPA score	0–1	9
	1.5–2.5	36
	3	6
	3.5–4	3

Abbreviations: SRS, stereotactic radiosurgery; SRT, stereotactic radiotherapy; RPA, recursive partitioning analysis; GPA, graded prognostic assessment; and

* Previous SRS or SRT to other tumors.

**Table 3.** Univariate analysis of prognostic factors affecting local control (LC), overall survival (OS), and freedom from distant brain failure (FDBF).

Factor (sort, *n*)	Rate at 12 months (*p* value)		

	LC	OS	FDBF
Age (≤65 *vs.* >65 years, 25 *vs.* 29)	82, 91 (0.2)	42, 61 (0.4)	66, 25 (0.1)
Gender (male *vs.* female, 31 *vs.* 23)	96, 76 (0.7)	49, 57 (0.3)	42, 40 (0.9)
Tumor number (1 *vs.* ≥2, 22 *vs.* 32)	100, 79 (0.04)	53, 51 (0.2)	58, 31 (0.4)
Tumor number (≤3 *vs.* ≥4, 42 *vs.* 12)	86, 90 (0.2)	56, 36 (0.08)	38, 46 (0.7)
GTV (<1 *vs.* ≥1 cc, 85 *vs.* 43)	90, 93 (0.8)	–	–
GTV (<2 *vs.* ≥2 cc, 100 *vs.* 28)	91, 89 (0.1)	–	–
GTV (<3 *vs.* ≥3 cc, 104 *vs.* 24)	91, 92 (0.3)	–	–
GTV (<5 *vs.* ≥5 cc, 112 *vs.* 16)	89, 100 (0.7)	–	–
GTV (<10 *vs.* ≥10 cc, 122 *vs.* 6)	90, 100 (0.9)	–	–
Cumulative GTV (<2 *vs.* ≥2 cc, 27 *vs.* 27)	45, 71 (0.7)	72, 31 (<0.0001)	41, 45 (0.9)
Cumulative GTV (<3 *vs.* ≥3 cc, 32 *vs.* 22)	52, 71 (0.8)	63, 37 (0.2)	41, 46 (0.6)

Extracranial disease (controlled *vs.* uncontrolled, 11 *vs.* 43)	86, 93 (0.2)	73, 34 (0.0002)	41, 46 (0.5)

Abbreviations: GTV, gross tumor volume; LC, local control; OS, overall survival; FDBF, freedom from distant brain failure, and controlled means that all extracranial diseases were in complete response.

**Table 4.** Multivariate analysis for prognostic factors affecting LC, OS, and FDBF.

Factor (sort, *n*)	*p* value, HR, 95% CI		
	LC	OS	FDBF
Tumor number (1 *vs.* ≥2, 22 *vs.* 32)	0.9, 1.1, 0.09–13	0.4, 0.7, 0.3–1.7	0.7, 1.2, 0.5–3.0
Cumulative GTV (<2 *vs.* ≥2 cc, 100 *vs.* 28)	0.9, 0.0, 0.0–1.5	0.6, 1.2, 0.5–2.9	0.4, 0.7, 0.3–1.7
Extracranial disease (controlled *vs.* uncontrolled, 11 *vs.* 43)	0.6, 0.5, 0.04–6.1	0.002, 0.2, 0.09–0.6	0.5, 0.7, 0.3–1.8

Abbreviations: GTV, gross tumor volume; LC, local control; OS, overall survival; FDBF, freedom from distant brain failure; HR, hazard ratio; and CI, confidence interval.

**Table 5.** Histology and tumor size.

Primary lesion	Histology	≤0.25 cc	>0.25 and ≤1 cc	>1 and ≤3 cc	>3 and ≤10 cc	>10 cc
Lung	Squamous	5	3	6	2	1
	Adeno	32	29	11	8	3
	Small cell	0	2	2	2	0
	PD	0	2	0	2	0
	UD	0	2	0	1	1

Liver	PD	1	1	0	1	0
Unknown	–	0	0	0	0	1
Colon	Adeno	0	0	0	1	0

Breast	Ductal	5	1	0	0	0
	Scirrhous	0	1	0	1	0

Uterus cervix	Squamous	1	0	0	0	0

Abbreviations: PD, Poorly differentiated; and UD, Undifferentiated.

**Table 6.** Studies on stereotactic radiotherapy for brain metastases.

	Minniti		Fahrig		Martens	Inoue	Narayana	This study
Patient No	206		150		75	145	12	54
Median size	1.9 cc (cumulative GTV)		6.1 cc (PTV)		1.5 cc (GTV)	6.9 cc (GTV)	3.5 cm (GTV)	1.9 cc (cumulative GTV)
Dose (Gy)/fraction	20/1, 18/1, (15–16)/1	35/5	35/7	40/10	Mainly 30/6, 35/7, 30/5	27/3	30/5	28/4, 28.8/4
BED_10_ (Gy)	–	40–49.6	43.8	56	–	42.8	40	47.6, 48.6
LC rate at 12 months (%)	92	(96)	(85)	(87)	52	(95.8)	70	91
Median OS (month)	14		15		9.1	7	8.5	7
Isodose line	87		–		–	60	100	80
≥Gr 3 acute toxicity (%)	5.8	0	0	0	–	6.2	Increased steroid use: 15	0
≥Gr 3 late toxicity (%)		22	7	0	1.3			0

Abbreviations: LC, local control; OS, overall survival; Gr, grade; and BED, biologically effective dose.
